# Long-Term Safety and Efficacy of Alginate-Based Serosal Reinforcement (SEAL-G/SEAL-G MIST) Following Colorectal Anastomosis: A Multicenter, Comparative and Retrospective Cohort Study

**DOI:** 10.3390/jcm15041448

**Published:** 2026-02-12

**Authors:** Fahim Kanani, Antonino Spinelli, Mordechai Shimonov, Husam Zbede, Nouha Hinnawi, Ron Lavy, Oded Zmora, Moshe Kamar

**Affiliations:** 1 Surgical Department, Wolfson Medical Center, Holon 5822012, Israel; shimonov.m@wmc.gov.il (M.S.);; 2 Department of Abdominal Organ Transplantation, Rabin Medical Center (Beilinson Campus), Tel Aviv University, Tel Aviv-Yafo 6997801, Israel; 3 Department of Biomedical Sciences, Humanitas University, Via Rita Levi Montalcini 4, Pieve Emanuele, 20072 Milan, Italy; antonino.spinelli@hunimed.eu; 4 IRCCS Humanitas Research Hospital, Via Manzoni 56, Rozzano, 20089 Milan, Italy; 5 Surgical Department, Shamir Medical Center (Asaf Harofeh), Be’er Ya’akov 70300, Israel; zbedehus@gmail.com (H.Z.); nouha05.06@hotmail.com (N.H.);

**Keywords:** anastomotic leakage, colorectal surgery, serosal reinforcement, alginate sealant, SEAL-G, long-term safety

## Abstract

**Background:** Anastomotic leakage (AL) remains a major complication following colorectal surgery (3–19% incidence, 6–39% mortality). SEAL-G/SEAL-G MIST are alginate-based sealants for anastomotic reinforcement. While short-term safety and efficacy feasibility have been established in a previous study, this study reports long-term results. **Methods:** A multicenter, retrospective and comparative study at three centers (Israel, Italy). Retrospective Treatment group: 79 patients from the original study treated with SEAL-G/SEAL-G MIST during elective colon cancer resection (2021–2023). Retrospective Control group: 86 comparative patients with standard technique. Primary endpoint: Incidence of long-term complications (adhesions, stenosis, stricture, obstruction) at 1 year. Secondary endpoints: Complications at 1–4 years and 30-day AL rate. Non-inferiority assessed via Farrington–Manning method (margin 0.10). **Results:** Mean follow-up: 3.3 ± 0.63 years (treatment) vs. 3.4 ± 1.10 years (control). Groups were comparable for demographics and surgical characteristics. Long-term complications at 1 year: 1.27% (1/79) vs. 2.33% (2/86); 90% CI for difference: −0.067 to 0.046, *p* = 0.0048 (non-inferiority confirmed). No stenosis or stricture occurred in either group. No additional complications emerged at 1–4 years in the treatment group. Thirty-day AL rate: 1.27% (1/79) vs. 5.68% (5/88); all subclinical leaks (Grade B, n = 4) occurred in controls. **Conclusions:** Serosal reinforcement with alginate-based sealants does not introduce device-related long-term complications following colorectal anastomosis. The favorable short-term safety profile extends to 2–4 years. These findings support the safety of alginate-based sealants as anastomotic adjuncts, consistent with the paradigm of leak containment and severity reduction.

## 1. Introduction

Anastomotic leakage (AL) remains the most consequential complication following colorectal surgery, with reported incidence rates of 3–19%, depending on anatomical location, and associated mortality reaching 6–39% [1,2]. Beyond the immediate clinical burden, AL substantially increases healthcare costs, prolongs hospitalization by a factor of 2.4–5.3, and negatively impacts oncological outcomes through delayed adjuvant therapy and increased local recurrence rates [1,3].

Over the years many factors have been attributed as risk factors for AL. Therefore, traditional approaches to AL have focused primarily on preventing leaks through technical optimization, patient selection, and perioperative care bundles with little success in overall AL prevention rates, as shown by large registries. Recent meta-analyses have demonstrated variable efficacy of preventive strategies: ICG fluorescence angiography reduces AL by approximately 55% (OR 0.45), reinforcement sutures by 58% (OR 0.42), and polyglycolic acid sheets by 72% (OR 0.28) [4]. However, emerging evidence suggests a paradigm shift: rather than exclusively targeting leak prevention, contemporary strategies increasingly emphasize leak containment and severity mitigation [4,5]. This conceptual evolution recognizes that while complete elimination of AL may be unattainable, transforming potential surgical failures into manageable clinical events represents a pragmatic therapeutic goal.

Serosal reinforcement using bioadhesive agents offers a mechanistic approach aligned with this containment paradigm. By providing reinforcement and a supplementary barrier at the anastomotic site, such devices may limit the consequences of clinical anastomotic dehiscence, potentially reducing the proportion of leaks requiring surgical reintervention. A recent 10-year retrospective analysis of serosal reinforcement demonstrated significant reduction in clinically significant leaks (ISREC Grade B + C: 1.1% vs. 5.3%, *p* = 0.03) without increase in long-term complications, including stenosis (1.1% vs. 0.6%), small bowel obstruction (1.7% vs. 1.2%), or cancer recurrence (4.4% vs. 3.5%) [5].

SEAL-G and SEAL-G MIST are alginate-based surgical sealants indicated for reinforcement and protection of gastrointestinal anastomoses. A prior prospective multicenter clinical study (2021–2023) evaluated short-term safety and 30-day anastomotic leak rates, demonstrating an overall leak rate of 3.1%, with only 0.6% requiring reoperation [6].

This multicenter, retrospective study was designed as a continuation of the original study, with follow-up at 2–4 years post-surgery. The primary objective was to determine whether SEAL-G/SEAL-G MIST introduces device-related long-term complications—specifically adhesions, stenosis, stricture, and bowel obstruction—compared to standard surgical technique alone.

## 2. Methods

### 2.1. Study Design and Setting

This observational, retrospective, comparative, and multicenter study was conducted in accordance with ISO 14155 [7] and Good Clinical Practice principles. The study was designed as a long-term continuation of a prior study that evaluated short-term safety and performance of SEAL-G/SEAL-G MIST in patients undergoing colorectal resection with primary anastomosis. Three centers from the original study were selected based on patient volume and ability to comply with follow-up procedures: Shamir Medical Center (Israel), Humanitas Research Hospital (Italy), and Wolfson Medical Center (Israel).

### 2.2. Participants

The treatment group comprised all patients (from the above-mentioned centers) who received SEAL-G or SEAL-G MIST serosal reinforcement during elective colon cancer resection with primary anastomosis in the original study. A comparable control group was retrospectively identified from patients who underwent similar procedures during 2021–2023 at the same institutions without sealant application (standard of care). Identical eligibility criteria were applied to both groups. No formal matching algorithm (propensity score, case–control, or frequency matching) was employed; comparability was assessed descriptively across baseline demographics and surgical characteristics. Both treatment and control group procedures were performed by the same surgical teams. Eligibility criteria for both groups included being aged 18–80 years, having undergone elective open or laparoscopic colon cancer resection with primary anastomosis, BMI ≤ 40 kg/m^2^, and ASA score ≤ 3. With respect to specific confounders, anastomotic level was controlled by excluding low anterior resection and anastomosis < 10 cm from the anal verge in both groups; no patient in either group received proximal fecal diversion (stoma-forming procedures were excluded). Intraoperative perfusion assessment (e.g., indocyanine green fluorescence angiography) was not systematically recorded in either group. Control group patients who received any anastomotic sealant or glue were excluded.

Anastomotic technique followed each center’s standard practice as previously described [6]. The majority of anastomoses were stapled: intracorporeal right colectomy anastomoses were constructed using linear staplers (side-to-side isoperistaltic technique), while left-sided and sigmoid anastomoses predominantly employed circular end-to-end staplers. Both treatment and control group procedures were performed by the same surgical teams at the same institutions during a comparable time period (2021–2023).

The final safety population included 165 subjects (treatment n = 79, control n = 86) who completed long-term follow-up.

### 2.3. Data Collection

Long-term follow-up was conducted between August and November 2025 via structured telephone questionnaires administered by site investigators, supplemented by medical record review. The questionnaire assessed the occurrence of long-term anastomotic complications, adhesions, stenosis, stricture, bowel obstruction, related hospitalizations, and reoperations. Follow-up was completed by 79/104 patients (76.0%) in the treatment group and 86 patients in the control group. Reasons for non-completion included loss to follow-up and death unrelated to anastomotic complications (Table 1). Site Principal Investigators conducted the telephone questionnaires and were not blinded to group assignment. Questionnaire responses were supplemented by review of medical records, hospitalization records, and standard-of-care colonoscopy and/or CT scan reports performed at approximately 1 year post-surgery for oncologic surveillance. The study sponsor performed independent data monitoring with source data verification against medical records. The mean follow-up interval was 3.3 ± 0.63 years for the treatment group and 3.4 ± 1.10 years for the control group (range 1.8–5.3 years).

Thirty-day anastomotic leak data for the treatment group were obtained from original study records; control group leak data were collected retrospectively from institutional medical records.

### 2.4. Outcomes

The primary safety endpoints were the incidence of long-term complications: adhesions, stenosis, anastomotic stricture, obstruction and reoperation rate due to anastomotic complications, at 1 year post-surgery. Secondary endpoints included the incidence of anastomotic complications, hospitalization, and reoperation at 1–4 years post-surgery, as well as 30-day anastomotic leak rate.

Anastomotic leaks were classified according to the International Study Group of Rectal Cancer (ISREC) grading system [8]: Grade A (requiring no active intervention, subclinical), Grade B (requiring active intervention without relaparotomy, subclinical), and Grade C (requiring relaparotomy, clinical).

### 2.5. Statistical Analysis

Non-inferiority of the treatment group compared to the control was assessed using the Farrington–Manning score method with a margin of 0.10. Two-sided 90% confidence intervals were calculated for between-group differences in proportions; a significant *p*-value indicates non-inferiority. Continuous variables were summarized as mean ± standard deviation, median, and range; categorical variables as counts and percentages.

### 2.6. Ethical Considerations

The study received ethics committee approval at all sites: Shamir Medical Center Helsinki Committee (ASF-0121-25), Wolfson Medical Center Helsinki Committee (WOMC-0119-25), and Lombardia Local Ethics Committee at Humanitas (No. 4581). The study was conducted according to the Declaration of Helsinki.

## 3. Results

### 3.1. Patient Disposition and Follow-Up

Of the 104 patients treated with SEAL-G/SEAL-G MIST in the original study, 79 (76.0%) completed long-term follow-up and were included in the safety analysis. Twenty-five patients were excluded due to loss to follow-up (n = 16) or death unrelated to anastomotic complications (n = 9). For the control group, 86 of 104 planned subjects (82.7%) completed follow-up; 15 were excluded due to loss to follow-up (n = 7) or death (n = 8). An additional two deceased control patients with available 30-day leak data from prior records were included in the FAS population for leak analysis (Table 1).

The mean follow-up interval was comparable between groups: 3.3 ± 0.63 years (range 2.3–4.8) for the treatment group and 3.4 ± 1.10 years (range 1.8–5.3) for the control group.

### 3.2. Baseline Characteristics

Demographic and surgical characteristics were comparable between groups (Table 2). The mean age was 67.0 ± 10.4 years in the treatment group and 66.8 ± 10.8 years in the control group. The mean BMI was 26.8 ± 4.07 kg/m^2^ and 26.6 ± 4.67 kg/m^2^, respectively. The majority of patients had an ASA score of 2 (65.8% treatment vs. 60.2% control).

Right colectomy was the most common procedure in both groups and was analyzed separately; see Section 3.

Right colectomy was the most common procedure in both groups (64.6% vs. 67.0%), followed by sigmoidectomy (16.5% vs. 17.0%) and left colectomy (13.9% vs. 12.5%). The laparoscopic approach was used in 88.6% of treatment group procedures and 80.7% of control group procedures.

### 3.3. Primary Safety Endpoints

The incidence of long-term anastomotic complications (adhesions, stenosis, stricture, or obstruction) at 1 year post-surgery was lower in the treatment group compared to in the control: 1/79 (1.27%) vs. 2/86 (2.33%). The 90% confidence interval for the difference (control minus treatment) was −0.0673 to 0.0461, with *p* = 0.0048, demonstrating non-inferiority of the treatment group (Table 3).

No cases of stenosis or stricture were observed in either group at any time point. Bowel obstruction occurred in 0 treatment patients and 2 control patients (2.33%). Adhesions were identified in one patient per group. Reoperation due to anastomotic complications within 1 year occurred in 0 treatment patients and 1 control patient (1.16%; 90% CI: −0.0648 to 0.0416).

### 3.4. Secondary Endpoints

#### 3.4.1. Long-Term Complications at 1–4 Years

Beyond 1 year post-surgery, no additional long-term anastomotic complications occurred in the treatment group. One control patient experienced adhesions at approximately 1.8 years post-surgery in the context of cancer recurrence, requiring multiple reoperations (Table 4). No hospitalizations or reoperations due to anastomotic complications occurred in either group during the 1–4 year interval.

#### 3.4.2. Anastomotic Leak Rate at 30 Days

The overall anastomotic leak rate within 30 days post-surgery was 1.27% (1/79) in the treatment group compared to 5.68% (5/88) in the control group (90% CI: −0.1010 to 0.0127) (Table 5). Clinical anastomotic leak (Grade C, requiring relaparotomy) occurred in one patient per group. Subclinical leaks (Grade B) occurred exclusively in the control group: four patients (4.55%) were managed conservatively with antibiotics and/or drainage. All observed anastomotic leaks in both groups were Grade B or C; no Grade A leaks were identified.

In a subgroup analysis restricted to right colectomy patients (n = 110), the 30-day anastomotic leak rate was 1.96% (1/51) in the treatment group versus 5.0% (3/60) in the control group. Long-term anastomotic complications occurred in 0/51 treatment patients versus 1/59 control patients.

Among the three patients with long-term anastomotic complications (adhesions/obstruction), two events occurred following laparoscopic procedures (one treatment, one control) and one following an open procedure (control), in the context of cancer recurrence requiring reoperation.

Find the details of anastomotic leak cases shown in Table 6, and the details of long term complicated cases in Table 7.

#### 3.4.3. Case Descriptions

Three patients experienced long-term anastomotic complications within the follow-up period. In the control group, a 74-year-old male who underwent laparoscopic sigmoidectomy developed bowel obstruction within 30 days, requiring reoperation during which adhesions were diagnosed. A second control patient, a 72-year-old male who underwent open right colectomy, developed cancer recurrence at 1 year with subsequent bowel obstruction, multiple hospitalizations, ileostomy creation, and adhesions identified at 1.8 years. In the treatment group, one patient, a 60-year-old male following laparoscopic left colectomy, had adhesions identified during liver resection for cancer recurrence at 7 months; colonoscopy at 3 years demonstrated normal anastomosis.

Regarding 30-day anastomotic leaks, one treatment group patient developed a clinical leak on postoperative day 3 due to technical error, managed with re-anastomosis. In the control group, one patient required reoperation with Hartmann’s procedure for a clinical leak on postoperative day 7. Four additional control patients developed subclinical leaks managed conservatively with antibiotics and/or drainage.

## 4. Discussion

### 4.1. Principal Findings

This multicenter, retrospective study evaluated the long-term safety profile of SEAL-G/SEAL-G MIST alginate-based sealants at 2–4 years following colorectal anastomosis. The primary finding is that serosal reinforcement with alginate-based sealants does not appear to introduce device-related long-term complications. The incidence of adhesions, stenosis, stricture, and bowel obstruction at 1 year was numerically lower in the treatment group compared to standard care (1.27% vs. 2.33%, *p* = 0.0048 for non-inferiority). Notably, no cases of stenosis or stricture were observed in either group throughout the entire follow-up period, and no additional anastomotic complications emerged between 1 and 4 years in patients who received sealant reinforcement.

These findings address an important gap in the clinical evidence for alginate-based anastomotic sealants. The original prospective study by Kamar et al. [6] demonstrated favorable short-term outcomes with an overall 30-day leak rate of 3.1% and only 0.6% requiring reoperation, but follow-up was limited to 30 days. The present study extends this evidence to a mean of 3.3 years, confirming that the early protective effect observed in the original cohort is not offset by delayed device-related complications.

A secondary observation of clinical relevance is the lower 30-day anastomotic leak rate in the treatment group compared to the retrospectively identified control cohort (1.27% vs. 5.68%). This comparison should be considered exploratory and hypothesis-generating, given the retrospective design and absence of formal matching. Nevertheless, the direction and magnitude of difference are consistent with the hypothesis that serosal reinforcement provides meaningful protection during the critical early healing period [4,5]. It is therefore suggested that alginate-based sealants may be effective at containing anastomotic defects, limiting potential free/uncontrolled leaks.

In a subgroup analysis restricted to right colectomy—the predominant procedure in both groups (~65%)—the direction and magnitude of the leak rate difference were consistent with the overall cohort (1.96% vs. 5.0%). This suggests that the observed patterns are not driven by procedure type distribution. However, the small non-right colectomy subgroups (28 per group) preclude assessment of left-sided anastomoses, where leak rates are known to be higher.

### 4.2. Comparison with Published Literature

The long-term safety profile observed in this study aligns closely with findings from other serosal reinforcement strategies. Kanani et al. [5] reported a 10-year experience with Hemopatch serosal reinforcement in 352 patients undergoing right colectomy. In that cohort, long-term complication rates were comparable between reinforcement and standard suture groups: stenosis occurred in 1.1% versus 0.6%, small bowel obstruction in 1.7% versus 1.2%, and cancer recurrence in 4.4% versus 3.5%, with no statistically significant differences [5]. The current study demonstrates a similar pattern: no stenosis or stricture in either group, obstruction in 0% versus 2.33%, and adhesions identified incidentally in comparable proportions when patients underwent reoperation for unrelated indications.

The SEAL-G/SEAL-G MIST appear to provide temporary mechanical support during the vulnerable healing period without leaving a permanent foreign body that might predispose to late complications. SEAL-G is gradually resorbed over 3–4 months through macrophage phagocytosis [6,9]. The device’s mechanism of action, which does not involve chemical or biological tissue interaction, together with its transient presence may be sufficient to protect the anastomosis during the anastomosis vulnerability period while avoiding the chronic inflammatory response associated with permanent implants [10].

### 4.3. The Paradigm Shift: From Prevention to Containment

These collective findings support an evolving conceptual framework in anastomotic protection. Traditional approaches have focused on leak prevention through optimization of surgical technique, patient selection, and perioperative care [11,12]. While these measures remain fundamental, accumulating evidence suggests that some degree of anastomotic imperfection is inevitable even under optimal conditions [4,5]. The systematic review by Kanani et al. [4] synthesized data from 30 high-quality studies and identified several effective preventive strategies—ICG fluorescence angiography (55% reduction, OR 0.45), reinforcement sutures (58% reduction, OR 0.42), and polyglycolic acid sheets (72% reduction, OR 0.28)—yet acknowledged that no single intervention eliminates anastomotic leakage entirely.

The paradigm shift toward leak containment and severity reduction offers a complementary strategy [4,5,13]. Rather than attempting to prevent all anastomotic failures, this approach aims to transform potential catastrophic dehiscence into a manageable perianastomotic abscess/collection, avoiding surgery. The clinical implications are substantial: Grade C leaks requiring relaparotomy carry mortality rates of 6–39% and dramatically increase morbidity, length of stay, and healthcare costs [6,14,15]. Grade A and B leaks, while not inconsequential, are associated with far better outcomes and can often be managed with antibiotics, drainage, or observation alone [8,16].

The data from both the current study and the Hemopatch series [5] support this containment hypothesis. In the Hemopatch cohort, no patients in the reinforcement group required relaparotomy for anastomotic leak compared to 28.6% in the standard suture group (*p* = 0.06) [5]. CT-confirmed leaks were significantly less frequent with reinforcement (5.0% vs. 13.5%, *p* < 0.01), suggesting that serosal coverage may seal minor defects before they become radiologically apparent [5].

This pattern—comparable rates of severe complications but reduced rates of clinically significant and subclinical leaks—is precisely what would be expected if serosal reinforcement functions primarily as a containment strategy. The sealant does not prevent the biological and mechanical factors that lead to anastomotic imperfection, but it may provide a secondary barrier that contains extravasation, reduces contamination, and allows minor defects to heal without clinical consequence [4,5,17].

### 4.4. Safety Considerations

A central concern with any anastomotic adjunct is the potential for device-related complications. Foreign-body reactions, chronic inflammation, and mechanical effects could theoretically increase rates of stenosis, stricture, adhesion formation, or bowel obstruction [18,19]. Cyanoacrylate-based adhesives, for example, have demonstrated effective sealing properties but can elicit significant foreign body reactions and inflammatory responses that may paradoxically impair healing [13,20]. The present study was specifically designed to address these concerns through long-term follow-up of alginate-based sealants, which function through physical adhesion rather than chemical bonding [9].

The results are reassuring. No cases of stenosis or stricture were observed in either group over a mean follow-up of 3.3–3.4 years. This finding is particularly relevant given that alginate-based sealants form a circumferential covering around the anastomosis, which might theoretically constrain luminal diameter during healing. The absence of stenosis suggests that the gradual resorption of the sealant occurs at an appropriate rate relative to tissue remodeling [6,9]. Similarly, the Hemopatch study reported stenosis rates of only 1.1% with no significant difference from the standard suture technique [5].

The sealant reinforces the serosal surface and provides a supplementary barrier at the anastomotic site. While the submucosa remains the primary strength layer of the anastomosis, per established surgical principles, the serosal covering serves to contain minor anastomotic defects during the critical healing period, potentially transforming clinically significant leaks into conservatively manageable events [6]. This containment mechanism is complementary to, rather than in conflict with, standard anastomotic construction techniques.

Adhesion formation was identified in one treatment patient and two control patients, all in the context of reoperation for cancer recurrence rather than primary anastomotic complications. The treatment group patient had adhesions noted incidentally during liver resection but demonstrated a normal anastomosis on colonoscopy at 3 years post-surgery. The published literature suggests that peritoneal adhesions develop after 67–93% of lower abdominal laparotomies, though only 15–18% require surgical re-intervention [21]. These findings suggest that adhesion rates are not increased by sealant application and that when adhesions do occur, they do not appear to compromise anastomotic integrity or function.

Bowel obstruction occurred in two control patients (2.33%) and in no treatment patients. While the numbers are small, this observation does not support concerns that SEAL-G or SEAL-G MIST serosal reinforcement might predispose to mechanical complications. The incidence of late-postoperative bowel obstruction after colorectal surgery is reported at 5–10%, with adhesions being the primary cause [22]. Both control patients who developed obstruction in the current study had confounding factors—one within 30 days of surgery related to the index anastomosis, and one in the context of cancer recurrence with tumor-related obstruction.

### 4.5. Comparison with the Original SEAL-G Study

This retrospective long-term follow-up study complements the original prospective study by Kamar et al. [6] but differs in several important methodological aspects. The original study was a prospective, single-arm, multicenter trial enrolling 160 patients at eight centers across Israel, Italy, and the United Kingdom, with 30-day follow-up for anastomotic leak and perioperative complications [6,23,24]. The current study is retrospective, includes a control group, and extends follow-up to 2–4 years, but includes only 79 treatment patients from three of the original eight centers.

The 30-day leak rate observed in the current treatment cohort (1.27%, 1/79) is numerically lower than in the original study (3.1%, 5/160). This difference is driven by the fact that only three centers out of five contributed data for the long-term follow-up, which may represent institutions with particular expertise or patient populations that differ from the broader original cohort. In the original prospective study [6], the mean length of hospital stay was 5.8 ± 3.7 days (5.1 ± 2.8 days for laparoscopic, 8.4 ± 5.5 days for open procedures, *p* < 0.001), with no 30-day readmissions or mortality. Length of stay was not a pre-specified endpoint in the current long-term follow-up study, which was designed to assess delayed anastomotic complications via telephone questionnaire and medical record review.

The control group leak rate of 5.68% in the current study is consistent with the published literature for colorectal anastomosis without reinforcement [4,25,26] and provides important context for interpreting the treatment group results. The original SEAL-G study lacked a concurrent control group, limiting conclusions about comparative efficacy [6]. The present data, while retrospective, suggest that the low leak rates observed with alginate-based sealants represent a genuine reduction compared to standard surgical technique rather than simply reflecting favorable patient selection or institutional expertise.

Importantly, the ideal anastomotic sealant should provide mechanical support without hindering physiological healing [27]. Unlike fibrin sealants, which have yielded inconsistent results in leak prevention [28,29], alginate-based sealants form a watertight barrier through physical adhesion to tissue irregularities without inducing chemical or biological interaction [6,9]. This mechanism may explain the favorable safety profile observed in both short-term and long-term follow-up.

## 5. Limitations

Several limitations warrant consideration when interpreting these findings. First, the retrospective design introduces potential for selection bias. Although both groups were drawn from the same institutions during the same operative period (2021–2023), with the same surgical teams performing procedures in both groups, and identical eligibility criteria were applied, the retrospective identification of the control group cannot exclude unmeasured confounders that may have influenced both group assignment and outcomes. Second, long-term follow-up relied on telephone questionnaires and medical record review, which may underestimate the true incidence of complications—particularly asymptomatic adhesions or mild stenosis that did not prompt medical evaluation. Diagnosing adhesions noninvasively remains challenging due to the lack of specific symptoms and imaging limitations [21]. Investigators conducting the telephone questionnaires were not blinded to group assignment. Although the primary endpoints are largely objective—requiring operative confirmation (obstruction), endoscopic confirmation (stenosis), or radiological/operative confirmation (adhesions)—interviewer bias cannot be entirely excluded. Asymptomatic adhesions or mild stricture not prompting medical evaluation would not have been captured; this limitation applies equally to both groups. Furthermore, perioperative outcomes, including length of hospital stay, were not systematically collected for the current cohort, as the study was designed specifically to evaluate long-term device-related safety. Third, the sample size is modest, limiting statistical power to detect small differences in rare outcomes. The low event rates, while clinically favorable, result in wide confidence intervals that preclude definitive conclusions about the magnitude of any treatment effect. Additionally, the predominance of right colectomy in both groups (approximately 65%) limits generalizability to left-sided and sigmoid anastomoses, where anastomotic leak rates are known to be higher. Future studies specifically powered for left-sided resections are warranted.

Fourth, the control group was identified retrospectively from institutional records and may not be fully comparable to the treatment group despite similar eligibility criteria. The higher proportion of open surgery in the control group (19.3% vs. 11.4%) is a potential confounder. For long-term adhesion-related complications, open surgery is associated with greater adhesion formation than laparoscopic approaches. However, two of the three observed long-term complications occurred after laparoscopic procedures, and the single open-surgery case (Cont_ASF-06) was driven by cancer recurrence rather than primary adhesive disease, suggesting that the surgical approach imbalance does not account for the observed long-term complication pattern. For the 30-day leak rate comparison, the direction of this bias disfavors the treatment group, as open surgery is associated with higher complication rates. Stratified analysis by surgical approach confirmed a consistent direction of the treatment–control difference within both laparoscopic and open subgroups. Multivariable adjustment was not performed due to insufficient event counts. Fifth, unmeasured confounders, including intraoperative perfusion assessment, steroid exposure, and nutritional status, were not collected and may have differed between groups. Although ASA score (≤3) and BMI (≤40) eligibility criteria partially constrain the range of comorbidity burden, these do not substitute for direct assessment of these risk factors. Sixth, this study includes patients from only three of the eight centers that participated in the original PMCF study, potentially limiting generalizability to other practice settings.

### Future Directions

The present findings support the continued investigation of alginate-based sealants for anastomotic reinforcement. Prospective randomized controlled trials with long-term follow-up would provide more definitive evidence of efficacy and safety. Such trials should be adequately powered to detect differences in clinically significant leak rates (Grade B + C) rather than overall leak incidence, consistent with the containment paradigm supported by current evidence [4,5].

Additionally, comparative studies examining different serosal reinforcement strategies—alginate-based, collagen-based, fibrin-based, and polyglycolic acid sheets—would help identify optimal approaches for specific clinical scenarios [4]. Patient selection criteria, including identification of high-risk subgroups most likely to benefit from reinforcement, warrant further investigation. Obesity has been identified as a strong independent risk factor for anastomotic leak (OR 29.697) [5], and such patients may derive particular benefit from serosal reinforcement. Finally, health economic analyses examining the cost-effectiveness of routine serosal reinforcement versus selective application in high-risk patients would inform clinical practice guidelines and resource allocation.

## 6. Conclusions

This multicenter retrospective study provides reassuring evidence that serosal reinforcement with SEAL-G/SEAL-G MIST alginate-based sealants does not introduce device-related long-term complications following colorectal anastomosis. Over a mean follow-up of 3.3 years, no cases of stenosis or stricture were observed, and rates of adhesions and bowel obstruction were not increased compared to standard surgical technique. These findings extend the favorable short-term safety profile reported in the original prospective single-arm PMCF study [6] to the long-term setting through retrospective comparative analysis.

The observation of reduced 30-day anastomotic leak rates in the treatment group, while requiring cautious interpretation given the retrospective design, is consistent with the hypothesis that serosal reinforcement may contain minor anastomotic defects and reduce the clinical consequences of anastomotic imperfection. This aligns with an emerging paradigm in anastomotic protection that emphasizes severity reduction alongside prevention [4,5].

Taken together with published data on other serosal reinforcement strategies, these results suggest that alginate-based sealants represent a safe adjunct to standard anastomotic technique. Serosal reinforcement does not appear to introduce additional risk but may provide meaningful clinical benefit in reducing clinically relevant anastomotic complications. Prospective randomized trials with long-term follow-up are warranted to confirm these findings and establish the role of alginate-based sealants in routine colorectal surgical practice.

## Figures and Tables

**Table 1:** Analysis populations.

Parameter	Total	Treatment Group	Control Group
Safety Analysis Set, n	165	79	86
Full Analysis Set (FAS), n	167	79	88
Follow-up duration, years			
Mean (SD)	3.4 (0.90)	3.3 (0.63)	3.4 (1.10)
Median	3.3	3.3	3.3
Min, Max	1.8, 5.3	2.3, 4.8	1.8, 5.3

**Table 2:** Patient demographics and surgical characteristics (FAS population).

Characteristic	Total (n = 167)	Treatment (n = 79)	Control (n = 88)
Age, years			
Mean (SD)	66.9 (10.6)	67.0 (10.4)	66.8 (10.8)
Sex, n (%)			
Male	97 (58.1)	46 (58.2)	51 (58.0)
Female	70 (41.9)	33 (41.8)	37 (42.0)
BMI, kg/m^2^			
Mean (SD)	26.7 (4.38)	26.8 (4.07)	26.6 (4.67)
ASA Score, n (%)			
1	24 (14.4)	8 (10.1)	16 (18.2)
2	105 (62.9)	52 (65.8)	53 (60.2)
3	38 (22.8)	19 (24.1)	19 (21.6)
Procedure Type, n (%)			
Right colectomy	110 (65.9)	51 (64.6)	59 (67.0)
Left colectomy	22 (13.2)	11 (13.9)	11 (12.5)
Sigmoidectomy	28 (16.8)	13 (16.5)	15 (17.0)
Anterior resection	3 (1.8)	2 (2.5)	1 (1.1)
Subtotal colectomy	3 (1.8)	2 (2.5)	1 (1.1)
Other	1 (0.6)	0 (0.0)	1 (1.1)
Surgical Approach, n (%)			
Laparoscopic	141 (84.4)	70 (88.6)	71 (80.7)
Open	26 (15.6)	9 (11.4)	17 (19.3)

**Table 3:** Primary endpoint: Long-term anastomotic complications at 1 year post-surgery (safety population).

Outcome	Treatment (n = 79)	Control (n = 86)	90% CI of Difference *	*p*-Value
Any complication, n (%)	1 (1.27)	2 (2.33)	−0.0673, 0.0461	0.0048
Adhesions, n (%)	1 (1.27)	1 (1.16)	−0.0555, 0.0575	—
Stenosis, n (%)	0 (0.00)	0 (0.00)	—	—
Stricture, n (%)	0 (0.00)	0 (0.00)	—	—
Obstruction, n (%)	0 (0.00)	2 (2.33)	−0.0765, 0.0300	—
Reoperation, n (%)	0 (0.00)	1 (1.16)	−0.0648, 0.0416	—

* Farrington–Manning non-inferiority test, margin 0.10. Significant *p*-value indicates treatment is non-inferior to control.

**Table 4:** Secondary endpoint: long-term anastomotic complications at 1–4 years post-surgery (safety population).

Outcome	Treatment (n = 79)	Control (n = 86)	90% CI of Difference
Any complication, n (%)	0 (0.00)	1 (1.16)	−0.0648, 0.0416
Adhesions, n (%)	0 (0.00)	1 (1.16)	−0.0648, 0.0416
Stenosis, n (%)	0 (0.00)	0 (0.00)	—
Stricture, n (%)	0 (0.00)	0 (0.00)	—
Obstruction, n (%)	0 (0.00)	0 (0.00)	—
Hospitalization, n (%)	0 (0.00)	0 (0.00)	—
Reoperation, n (%)	0 (0.00)	0 (0.00)	—

**Table 5:** Secondary endpoint: anastomotic leak rate within 30 days post-surgery (FAS population).

Outcome	Treatment (n = 79)	Control (n = 88)	90% CI of Difference
Overall leak rate, n (%)	1 (1.27)	5 (5.68)	−0.1010, 0.0127
Clinical leak (Grade C), n (%)	1 (1.27)	1 (1.14)	—
Subclinical leak (Grade B), n (%)	0 (0.00)	4 (4.55)	—

**Table 6:** Details of anastomotic leak cases (FAS population).

Subject ID	Group	POD	Clinical Presentation	Management	Leak Grade
ASF-051	Treatment	3	Technical error	Re-anastomosis	C (Clinical)
Cont_ASF-21	Control	7	Leak	Hartmann’s procedure	C (Clinical)
Cont_ICH-20	Control	2	Ileus, CRP ↑, CT: Free air	Antibiotics	B (Subclinical)
Cont_ASF-24	Control	4	Fever, CT: Free fluids	Antibiotics	B (Subclinical)
Cont_ASF-50	Control	22	Wound infection, fistula	Antibiotics	B (Subclinical)
Cont_ASF-27	Control	7	Leak	Drain + Antibiotics	B (Subclinical)

POD, postoperative day; CRP, C-reactive protein; CT, computed tomography.

**Table 7:** Details of long-term complication cases (safety population).

Subject ID	Group	Age/Sex	BMI	Procedure	Complication	Time Post-Op	Management
Cont_ICH-06	Control	74/M	24.5	Lap sigmoidectomy	Obstruction, adhesions	<1 year	Reoperation
Cont_ASF-06	Control	72/M	25.4	Open right colectomy	Obstruction, adhesions *	1 year, 1.8 years	Multiple surgeries, ileostomy
ICH-014	Treatment	60/M	24.1	Lap left colectomy + SEAL-G MIST	Adhesions ^†^	7 months	None (found during liver resection)

* In context of cancer recurrence. ^†^ Colonoscopy at 3 years: normal anastomosis.

## Data Availability

The datasets generated and analyzed during the current study are available from the corresponding author upon reasonable request.

## Abbreviations

The following abbreviations are used in this manuscript:

AL	Anastomotic Leakage
ASA	American Society of Anesthesiologists
BMI	Body Mass Index
CI	Confidence Interval
CRP	C-Reactive Protein
CT	Computed Tomography
ERAS	Enhanced Recovery After Surgery
FAS	Full Analysis Set
ICG	Indocyanine Green
ISREC	International Study Group of Rectal Cancer
LAR	Low Anterior Resection
LOS	Length of Stay
PMCF	Post-Market Clinical Follow-up
POD	Postoperative Day
RCT	Randomized Controlled Trial
SBO	Small Bowel Obstruction
